# Utilizing Pyrolysis–Gas Chromatography/Mass Spectrometry for Monitoring and Analytical Characterization of Microplastics in Polychaete Worms

**DOI:** 10.3390/polym14153054

**Published:** 2022-07-28

**Authors:** Sabiqah Tuan Anuar, Raad Shaher Altarawnah, Ahmad Ammarluddin Mohd Ali, Bai Qin Lee, Wan Mohd Afiq Wan Mohd Khalik, Ku Mohd Kalkausar Ku Yusof, Yusof Shuaib Ibrahim

**Affiliations:** 1 Microplastic Research Interest Group (MRIG), Faculty of Science and Marine Environment, Universiti Malaysia Terengganu, Kuala Nerus 21030, Terengganu, Malaysia; wan.afiq@umt.edu.my (W.M.A.W.M.K.); kukautsar@umt.edu.my (K.M.K.K.Y.); 2 Faculty of Science and Marine Environment, Universiti Malaysia Terengganu, Kuala Nerus 21030, Terengganu, Malaysia; raedshaher01@gmail.com (R.S.A.); ammarali69.com@gmail.com (A.A.M.A.); 3 ALS Technichem (M) Sdn. Bhd., Wisma ALS, No. 21, Jalan Astaka U8/84, Bukit Jelutong, Shah Alam 40150, Selangor, Malaysia; lee.baiqin@alsglobal.com

**Keywords:** South China Sea, pollution, Py-GC/MS, fragmentation and degradation, mechanism

## Abstract

Microplastics (the term for plastics at sizes of <5 mm) might be introduced into the environment from domestic or agricultural activities or from the breakdown of plastic pieces, particles, and debris that are bigger in size. Their presence in the aquatic environment has caused accumulation problems, as microplastics do not easily break down and can be digested by some aquatic organisms. This study was conducted to screen and monitor the level of microplastic pollution in polychaete worms using pyrolysis–gas chromatography/mass spectrometry (Py-GC/MS). The study was conducted in Setiu Wetlands, Malaysia from November 2015 to January 2017 at five-month intervals and covered all monsoon changes. Results from physical and visual analyses indicated that a total number of 371.4 ± 20.2 items/g microplastics were retrieved from polychaete for all seasons, in which, the majority comprised transparent microplastics (49.87%), followed by brown with 138.3 ± 13.6 items/g (37.24%), 21.7 ± 1.9 items/g for blue (5.84%), and 12.9 ± 1.1 items/g for black (3.47%), while the remaining were green and grey-red colors. Statistical analysis using Kruskal–Wallis showed insignificant differences (*p* > 0.05) between the sampling station and period for the presence of a microplastics amount. Most of the microplastics were found in fiber form (81.5%), whereas the remaining comprised fragment (18.31%) and film (0.19%) forms. Further analysis with Py-GC/MS under a selective ion monitoring mode indicated that pyrolytic products and fragment ions for a variety of polymers, such as polyvinyl chloride, polypropylene, polyethylene, polyethylene terephthalate, polyamide, and polymethylmethacrylate, were detected. This study provides an insightful application of Py-GC/MS techniques for microplastics monitoring, especially when dealing with analytical amounts of samples.

## 1. Introduction

The ever-increasing human population has made a high demand for resources and living space, which has resulted in the expansion of terrestrial urban areas and subsequently intruded the coastal territory. The construction of buildings, the constant generation of municipal wastes, and the unearthing of natural resources has not only brought ecological changes, but has also negatively affected many organisms living therein through pollution, food scarcity, poor reproduction, and alternative predation [1,2]. As a result, these organisms are forced to change their routine and perquisites to cater for the rapid changes in their surroundings. As such, hydrocarbon-based contamination is threatening both aquatic and terrestrial life forms in human-infringed areas; ultimately, these contaminations have caused a species-wise population reduction, especially among less tolerant organisms [3,4]. The plastic derived from the industrial synthesis of natural gas and crude oil refining also represents another source of contamination/pollution that requires immediate attention [5,6]. For instance, the plastic wastes resulting from human-used products such as toothpaste, cosmetics, clothing, and plastic packaging are usually identified as microplastics due to their small size (1–5 mm) [7,8]. Among others, polystyrene (PS), polyethylene (PE), polypropylene (PP), polyethylene terephthalate (PET), polyvinyl chloride (PVC), nylon, and polyamides (PA) are some of the examples of the most abundant microplastics found in the coastal water [9].

The biological interaction between microplastics and biota is crucial to understanding the movement, impact, and fate of microplastics in the environment. Several controlled laboratory and/or field studies have been conducted to clarify the impact of this problem. Recent attention has been focused on lower trophic level organisms, as these marine invertebrates have significant effects on the introduction of microplastics into the food chain [10]. Recent studies revealed that an excessive amount of microplastics has been found in the tissue and gastrointestinal tract of small marine deposit feeders and scavengers [11,12,13] and respiratory and digestive tracts of crustaceans [14,15]. These microplastics were also detected in the body of larger predators after consuming lower trophic animals [16,17]. As a result, these higher trophic predators will suffer from physical blockage at their alimentary and digestive tract and will be poisoned after the digested plastic leaches into their digestive system and adsorbs on the organ interior lining [18]. In addition, the direct ingestion of microplastics by fish in riverine areas [17], as well as in estuaries [19], was also reported. This could then trigger major environmental and health issues [20,21].

Despite microplastic pollution being an emerging field of study, the impacts of microplastics on human health and the environment are yet to be fully discovered. Hence, the monitoring and identification of microplastic pollution is urgently needed prior to the development of efficient treatment strategies. As such, standardized field methods for collecting microplastics in sediment, sand, and surface-water samples and a novel analysis technique for the identification of microplastics have been developed and continue to be optimized. In due course, a global comparison of the amount of microplastics released can be carried out with field and laboratory protocols to elucidate the final distribution, impacts, and fate of microplastics [22]. Two innovative analysis techniques have been developed for the determination of microplastics in complex environmental samples using pyrolysis–gas chromatography/mass spectrometry (Py-GC/MS) and thermal desorption–gas chromatography/mass spectrometry (TD-GC/MS). The scientific and practical challenge of detecting microplastics in the target environment in a rapid manner can be resolved by thermogravimetric analysis with the mass spectrometry method. Using Py-GC/MS and TD-GC/MS, the rapid identification and quantitative determination of most thermoplastic polymers and elastomers is possible [23,24,25] with a limited sample size; for instance, in polychaete worms, the composition of the plastic material can be obtained via the Py-GC/MS. This study aims to investigate and monitor the pollution level of microplastics in Setiu Wetlands, Terengganu, Malaysia using Py-GC/MS, in which, to our knowledge, this is the first study in Malaysia to utilize the technique for microplastics identification.

## 2. Materials and Methods

### 2.1. Sampling Site and Sample Collection

**Figure 1:**
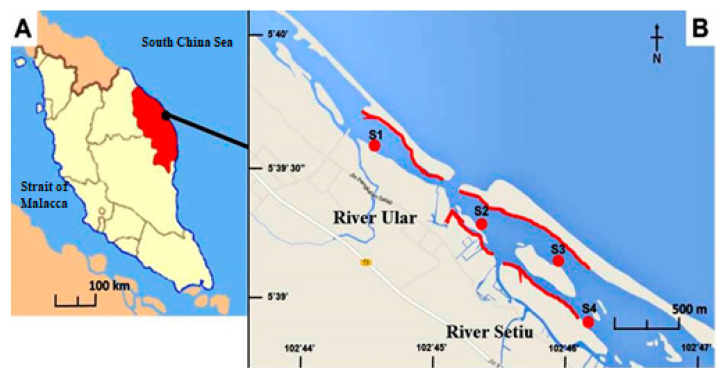
Map showing (**A**) Setiu Wetlands location at the east coast of peninsular Malaysia (facing South China Sea), and (**B**) Sampling stations S1–S4 at Setiu Wetland, Malaysia.

Setiu Wetland comprises 23,000 ha terrestrial and aquatic environments that experience hot and humid climate intervals, with air temperatures ranging between 28 °C and 33 °C, seasonal salinity shifts from 3–33%, and annual rainfall between 2000 and 4000 mm [26,27]. This large ecological system has freshwater and peat swamps that host riparian forests and mangrove–mangrove-associated vegetation along its riverbanks, grass beds, and sandy beaches, which also house diverse macrobenthos and invertebrates [28,29]. Four sampling sites at Setiu Wetlands were selected according to the preliminary investigation of polychaete distribution. Each of the sampling sites were coordinated and illustrated as shown in Table 1 and Figure 1, respectively. The source of fresh water in these areas is streamed from River Setiu and River Ular (Figure 1). These areas are covered by tidal action and wind-driven currents from mangrove trees (i.e., *Nypa fruiticans* and *Avicennia* sp.), which act as a shelter and dominate sand islets. On the other hand, marine water is channeled into the estuary of the South China Sea [30]. The input into the Setiu Wetlands system is usually retained for long periods during low tide because it is an almost closed system with only a single opening to the sea that is <20 m apart.

The sampling was performed four times from November 2015 until January 2017 in Setiu Wetlands, Terengganu, Malaysia, following the protocol of Hamzah et al. [11]. The field visits were conducted at four sites (i.e., three in Ular River and marked as S1–S3, and one in Setiu River and marked as S4, Figure 1). Each transect of 400 m^2^ (20 m × 20 m) was set and had a 200 m interval before the next station, as illustrated by the red lines in the map. Sampling was conducted during the intermediate tide (1.2–2.0 m) by searching for polychaete worms (*Namalycastis* sp.) at the marked *Nypa fruticans* (nypa palm) vegetated riverbanks. This species is among the most abundant organisms in the benthic community in the mangrove area of Setiu Wetlands. These worms have special ability to adapt to their environment, thus allowing them to structure the dynamics of their surroundings and feed on rotten nypa palm [29,31]. During the sample collection, encrusted decaying pieces of the plants (such as fronds and roots) were carefully sectioned to retrieve the polychaete worms using bare hands. A total of 160 individuals was collected by hand, and the site was marked for re-visiting using a portable handheld global positioning system (Garmin GPSMAP 78S GPS, Garmin Ltd., Olathe, KS, USA). The polychaetes collected from each site were kept in a glass container and stored in an icebox for laboratory analysis.

### 2.2. Extraction and Isolation of Microplastics

Ten polychaetes with approximate similar size and weights were pooled (*n* = 10, weight~10 g) from each site and added to 10 mL of 10 M NaOH in a digestion tube. Data from the physical analysis of microplastics (sorting and isolation) were expressed as microplastics item/g of the polychaete, since the polychaete was pooled based on different sampling stations and periods during the digestion process. The digestion was conducted at 60 °C for 48 h. After the digestion process, the brown-translucent solution was filtered using a vacuum pump with cellulose nitrate filter paper (Whatman 0.45 µm pore size, Cytiva, Marlborough, MA, USA) before being placed in a petri dish and dried in a desiccator. Physical identification of microplastics was conducted using the standardized size and color-sorted system according to the characterization protocol provided by Hidalgo-Ruz et al. [32]. The sample was sorted into three groups: fiber, fragment, and film, with various colors of brown (Br), transparent (T), red (R), blue (B), grey (Gy), black (Bl), and green (Gn) using dissecting microscope (Olympus CX21 8X-56X Magnification, Olympus Corp., Tokyo, Japan). All sorted samples were collected using a fine-tip stainless steel forceps and kept in wide-neck glass bottles (McCartney, Fisher Scientific, Waltham, MA, USA) containing 10 mL of filtered Milli-Q water.

### 2.3. Chemical Analysis for Microplastic Identification Using Py-GC/MS

The microplastics samples were analyzed via Py-GC/MS technique. This technique combines the process of pyrolysis, which is the degradation of high molecular polymer into smaller organic compounds by heating in a pyrolysis chamber (Frontier Lab. Ltd., Koriyama, Japan) in the absence of oxygen. The organic compounds were separated via the gas chromatography technique (GC 6890 N, Agilent Tech., Santa Clara, CA, USA). In brief, the GC is equipped with a pre-column (Ultra Alloy*-5, 30 m × 0.25 mm × 0.5 µm) and column (Ultra Alloy*-50, 2 m × 0.25 mm × 1.0 µm, Frontier Lab. Ltd., Koriyama, Japan) and characterized using mass spectrometer (MS 5973 N, Agilent Tech., CA, USA). For the analysis using Py-GC/MS, 20% of the microplastics item (for each station and period) was considered by adding 4 mg of calcium carbonate powder (Frontier Lab, Japan), and was derivatized with 5 µL 25% tetramethylammonium hydroxide (TMAH) solution in methanol (Sigma Aldrich, Petaling Jaya, Malaysia) after being filtered using an aluminum oxide filter prior to the pyrolysis. The temperature programs were developed and optimized according to the manual from the supplier of pyrolyzer, Frontier Laboratories Ltd. [33], whereas the selection of characteristic ions was following Fisher and Scholz-Bottcher [34].

In brief, after the pyrolysis cup was dropped into the pyrolysis chamber, the temperature of the pyrolysis furnace was maintained at 400 °C for 10 min. The transfer line was heated and maintained at 300 °C. The initial oven temperature of the GC was set at 40 °C and held for 2 min, then ramped to 280 °C at 20 °C/min and held for 10 min. Finally, the oven was ramped to 320 °C at 40 °C/min and held for 15 min. The inlet was set up with a split mode ratio of 50:1 and a constant pressure of 150 kPa by helium gas. Mass spectrometry was set to run with scan mode in the range between 29 and 350 amu. The MS scan rate was set at *n* = 2, equivalent to 4 scans/s. Subsequently, a set of the calibration curve was prepared based on twelve common polymers, including polyethylene (PE), polypropylene (PP), polystyrene (PS), polycarbonate (PC), polyvinyl chloride (PVC), polyurethane (PU), polyethylene terephthalate (PET), acrylonitrile butadiene styrene (ABS), styrene-butadiene (SBR), and polymethyl methacrylate (PMMA), and polyamides such as nylon-6 (N-6) and nylon-6,6 (N-66). Quantitation of these polymers was performed referring to the selected pyrolytic products (Table 2).

Additionally, microplastics calibration standard set was purchased from Frontier Lab (Frontier Lab. Ltd., Koriyama, Japan). Three-point calibration curve was created by the microplastics calibration standards containing calcium carbonate. In brief, 0.4 mg, 2.0 mg, and 4 mg of calibration standard were weighted precisely in the pyrolysis cup before 5 µL of 25% TMAH in methanol solution was added into the pyrolysis cup and left at room temperature for 20 min before the analysis. Analysis of calibration curve is as per described in Section 2.3. The R^2^ for built calibration is between 0.96 and 0.99 (Supplementary Figure S1).

### 2.4. Multivariate and Statistical Analysis

The statistical analyses were performed using OriginPro version 9.1 software (Origin Lab Corp., Northampton, MA, USA) and R Software (R development team, Version 1.4.1). The abundance of microplastics was evaluated using Kruskal–Wallis (post hoc: Bonferonni correction) subjected to a non-parametric dataset for various types and colors between the station and period of the study. The significance level was set at *p* < 0.05. The results obtained are presented in the form of a dendrogram graph, with 3 clusters of color following the number of the collected color sample within the sampling time. Box plot analysis was used to compare the shape distribution of the samples.

### 2.5. Quality Assurance and Control

Precautions were taken to prevent microplastics contamination during sample collection in the field and analysis in the dedicated clean room of the Microplastic Research Interest Group (MRIG) laboratory. Cotton lab gowns and single-use latex gloves were worn during all procedures of the experiment. The work surfaces were carefully washed with 80% filtered ethanol prior to the start of all procedures and during the experiment period. Glassware and instrumentation were washed with filtered Milli-Q water (Milli-Q water underwent double filtration with 1.2 µm Whatman GF/C filter paper prior usage) and rinsed three times with 80% ethanol. In addition, procedural blanks and control filter papers were first inspected by dissecting microscope (Olympus SZX-ZB7, Olympus Corp., Tokyo, Japan) and later thoroughly analyzed to detect any contamination of microplastics from the laboratory surroundings and during sample handling [11].

## 3. Results and Discussion

**Figure 2:**
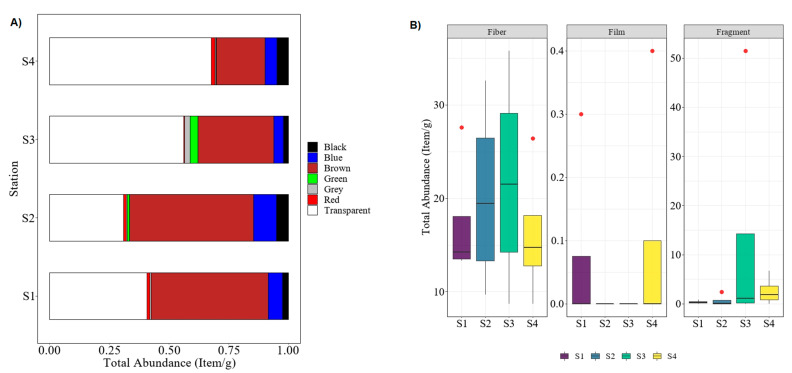
Percentage abundance of microplastic found in polychaete of sampling stations S1–S4: (**A**) based on color, (**B**) based on shape.

Originated from synthetic polymers, microplastics (size < 5 mm) can present in various shapes, sizes, and colors [22,35]. In this study, analyses of microplastics were separated into two parts. Firstly, physical and visual analysis, and secondly, polymer identification by chemical analysis. In the first part, the color of microplastics was sorted, followed by their type (i.e., fiber, fragment, and film). From the physical analysis, a total of 371.4 ± 20.2 items/g microplastic particles was extracted in collected polychaetes from four sampling stations. From that data, 185.2 ± 24.7 items/g are transparent microplastics (49.87%), followed by brown with 138.3 ± 13.6 items/g (37.24%), 21.7 ± 1.9 items/g for blue (5.84%), 12.9 ± 1.1 items/g for black (3.47%), 1.53% for green, 1.08% for grey, and, lastly, around 0.97% for red samples. The Kruskal–Wallis test showed that the total microplastics abundance between sampling stations was insignificantly different: *H*(3) = 1.51, *p* = 0.68. When comparing the sampling stations (Figure 2A), S1 and S2 indicated that brown microplastics were dominantly found in collected worms, followed by transparent, blue, black, and other colors of microplastics. However, in sampling sites of S3 and S4, the transparent color is the most dominant color among the microplastic found in polychaete samples, followed by brown, blue, black, and others.

Between stations, polychaete samples from S3 recorded the highest number of microplastics (141.2 items/g, 38.02%), while the total abundance for the remaining sampling stations (S1, S2, and S4) was 19.1–22.6%. Since S3 is located on the depositional bank of the terrestrial area (low island) and near the mouth of the Ular River, it is thus susceptible to the wash-over pollution from the river downstream and the terrestrial area, as well as the tidal influx activity of the sea that brings marine debris and microplastics to this area [35,36,37]. Additionally, the other reason for S3 becoming the most polluted area (in terms of ingested microplastics by polychaetes) could be due to its geographical area that is sheltered by the small island and near the closed inlet; thus, the ocean movement might be relatively slow in that area and subsequently cause an accumulation of microplastics waste. Therefore, the population of polychaetes in this area consumed more microplastics compared to other areas, which corroborated the findings reported by [11]. On the other hand, previous studies have suggested that the different amounts of microplastics collected at the sampling stations might be affected by the movement of the ocean or winds [30,38]. In this study, three types of microplastics were identified by the physical analysis, namely fiber (thread), fragment, and film, as specified by the boxplot analysis in Figure 2B. Of the total microplastics, 81.5% were fiber, followed by 18.31% in the form of fragments, and only 0.19% were film form. This finding was similar to the previous studies conducted by Su et al. [39] and Huang et al. [40]. Both studies indicated that more than 70% microplastics were found in the form of fiber, and the fewest microplastics were found in the form of film, which concurred with the findings obtained in this study. Generally, the fiber-type of microplastics are derived from the decomposition of synthetic fishing gears such as lines, nets, and ropes [18]. Since major economic activities at the Setiu Wetlands were agriculture-based, the pollution might therefore be sourced from these activities.

**Figure 3:**
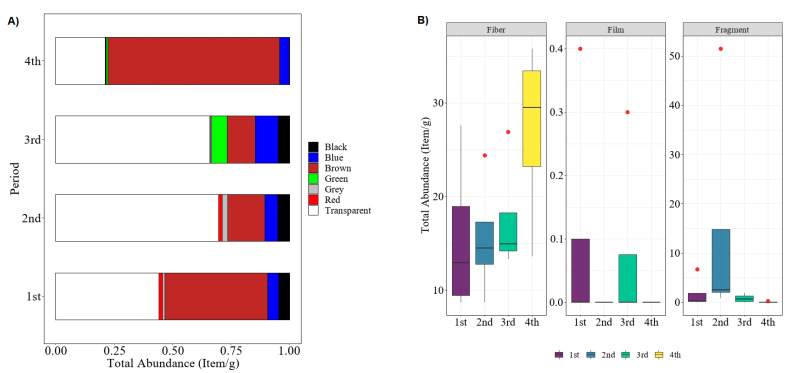
Total abundance of microplastic found in polychaete of sampling period 1 to 4: (**A**) based on color, (**B**) based on shape.

Meanwhile, Figure 3 indicates the total abundance of microplastics in polychaetes in four different sampling periods of November 2015 (1st), March 2016 (2nd), August 2016 (3rd), and January 2017 (4th) in term of colors (Figure 3A) and shapes (Figure 3B). During the first sampling period, the northeast monsoon was blown in the Setiu Wetlands and caused a heavy rainy season from November to January [41]. Generally, the movement of pollution agents into river streams was positively affected by the amount of rainfall. Therefore, when sampling activity happened in the second sampling period in March (post–northeast-monsoon), the number of microplastics increased by approximately 41.62% compared to the first sampling period. Interestingly, the most abundant microplastics found were transparent during the second and third sampling periods, while the brown microplastics became dominant when it was close to the northeast monsoon season. However, Kruskal–Wallis confirmed that there was an insignificant difference in terms of the total microplastics abundance between the sampling period, even with the variation in specific color observed: *H*(3) = 0.86, *p* = 0.834.

Similar to the previous findings by other researchers, the fiber type of microplastics represents the most common shape found in the polychaete samples [11,42] (Figure 3B). This could be attributed to the feeding behavior of the polychaete itself. This species tends to mistakenly ingest fiber microplastics since it can be similar to their feed, such as *Nypa fruticans* fibers. The discoloration of microplastics could be suggested as being due to the oxidation of microplastic-associated compounds either caused by environmental (e.g., weathering, chemical degradation) or biological (e.g., digestive environment of polychaete gut) factors. Polychaetes usually dominate the bottom community in open water, thus making them important for bioturbation. This organism can break down, deposit, and incorporate organic matter to recycle nutrients in their substrata [43,44]; ruling out pollutants such as microplastics during this process is inevitable. Previously, polychaetes have been used as a bioindicator for environmental pollution, especially for heavy metals and other toxic substances [45,46,47]. Additionally, the occurrence of the fiber type of microplastics in the estuarine ecosystem can be caused by both terrestrial activities and hydrological factors; since the size of microplastics is very small, they can easily get into the food chain and be consumed directly by the polychaetes in the ecosystem [11,42,47].

The next analysis for the microplastics was the chemical characterization via pyrolysis-GC/MS. Theoretically, this technique required the pyrolysis of microplastics at a high temperature, and the volatiles were separated using gas chromatography, finally detected, or analyzed via mass spectrometry. Table 2 summarizes the fragment ion data used for this technique, which include the pyrolytic products and *m*/*z* ratio that were selected for quantitative purposes, while Table 3 shows the polymer found using the analysis.

Py-GC/MS is a well-established separation technique for the investigation of volatile and non-volatile compounds. Py-GC/MS has been frequently applied in the microstructural characterization and identification of synthetic polymers and copolymers in many industrial applications [48,49]. Chemical additives such as organic plastic additives (OPAs) are usually added to modify and improve the physical, chemical, thermal, mechanical, and electrical properties of the polymers during the manufacturing process [50]. This chemical additive is available in small, volatile, and semi-volatile molecules, or large and less or non-volatile compounds. As such, pyrolytic products for PE were successfully identified (i.e., tetradecadiene with characteristic and sub-characteristic ions of 82 *m/z* and 294 *m/z*), respectively [51]. For PP, the decomposition fragment that could be used for this polymer identification is 2,4-dimethyl-1-heptene (126 *m/z*); whereas the presence of styrene trimer (91 *m/z*), naphthalene (128 *m/z*), and benzene (77 *m/z*) could be used for PS and PVC, respectively. Additionally, 2,2-bis(4′methoxy-phenyl) propane and 4-isopropenylphenol were identified as a decomposition fragment for PC [25,51,52].

**Figure 4:**
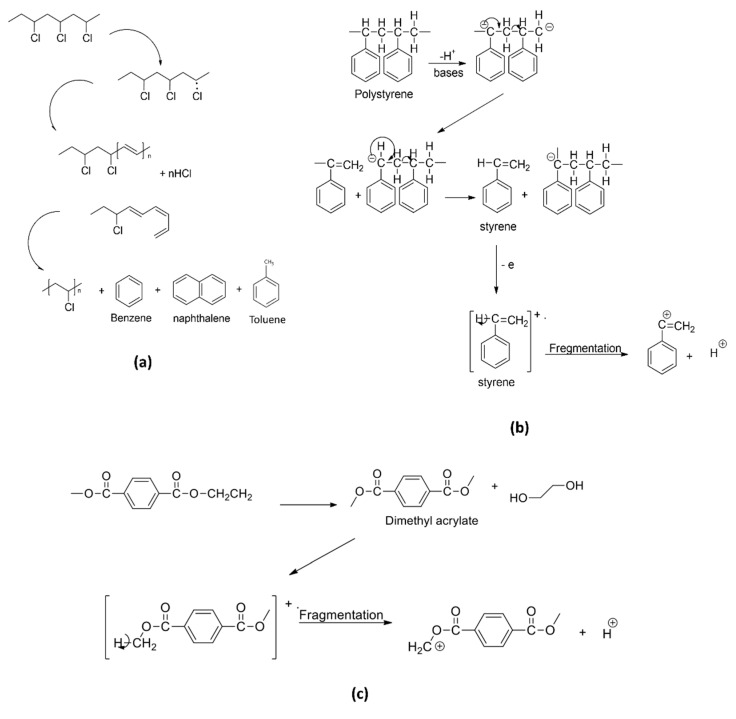
Simplification of degradation mechanism of common polymers into respective monomers: (**a**) PVC, (**b**) PS, and (**c**) PET.

**Figure 5:**
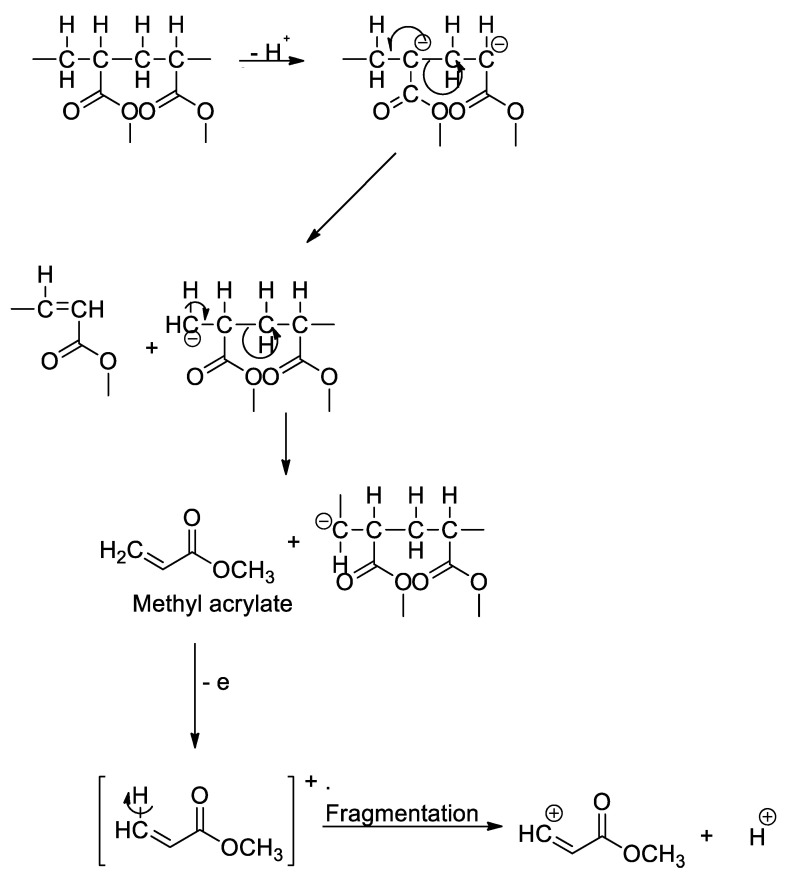
Fragmentation mechanism of PMMA into methyl acrylate monomer.

ε-Caprolactam, a monomer in the production of nylon-6, was used in the polyamide identification, in which, this fragment yielded a molecular weight of 133 *m/z*. For PET and PMMA identifications, the pyrolytic products benzophenone (182 *m/z*), primarily used in the manufacturing of PET fiber, film and container plastics, and methyl methacrylate (100 *m/z*), commonly used in the paint industry and as rheology modifiers, were used to identify the respective polymers. Examples of fragmentation mechanisms for PVC into naphthalene and subsequently into benzene or toluene monomer (a), PS into styrene monomer (b), and PET into dimethyl terephthalate (c) are illustrated in Figure 4 [53,54,55]. These mechanisms show the possible degradation pathways of the microplastics in the environment. For instance, PVC contains a high abundance of chlorine in the structure, thus leading to the formation and leachate of other chlorinated hydrocarbons when being induced by hydrolysis, photooxidation, and thermal degradation, which continuously happen in the environment. It will later be fragmented into the polyene chain and other PAHs derivatizes (e.g., toluene, benzene, and naphthalene) via random bond scission [54], as observed in the thermoanalysis of this polymeric material. Additionally, the possible fragmentation mechanism of PMMA into methyl methacrylate is illustrated in Figure 5. Similar to the industrial modification of PMMA into the monomers, the physical, biological, chemical, and thermal modifications occurred in the environment when the polymers were subjected to UV light, thermal changes, weathering, mechanical abrasion, and fragmentation. During the degradation process, the polymeric PMMA will lose its H^+^, which leads to the bond fragmentation to a smaller unit of monomers, such as methyl acrylate, as observed in the pyrolysis-GC/MS analysis.

Py-GC/MS could undoubtedly be used to characterize and quantify the relative mass content of microplastics in environmental samples with >50 µg/L (or ppb when describing the environmental contaminant concentrations) via the determination of specific polymeric decomposition products, independent of the natural organic and inorganic matrix [34]. It is noteworthy to state that, in our case, no additional preparation steps were needed if the homogenous sample size was used. Nonetheless, this technique can be applied to different matrices [56] and can allow for the quantification of the analytical size of samples. In this study, some of the samples were quantitatively measured in the range of 60–90 µg/L, while the rest were also detected despite being below the calibration limit. This, however, limits the information about the concentration of each polymer, which will need to be optimized in future work.

Nevertheless, based on the findings obtained (Table 3), PVC was found in all samples collected from all sampling locations. This contradicts PC, which has not been detected. PVC has a higher density than water (~1.38 g/cm3); thus, this polymer is susceptible to being deposited in the riverbed of the mangrove vegetation area during the tidal changes [11,57]. The high availability of this type of microplastic in such an ecosystem will lead to indirect or direct ingestion by the polychaetes. Additionally, both PE and PP were also identified in most of the polychaete samples, followed by PA, PET, and PMMA. The abundance of lower-density polymers found among the polychaete samples could be attributed to their ability to be transported by river flow and seawater influx around the estuary area (e.g., Setiu Wetlands) [19,30,40]. Notwithstanding, the molecular information on the polymer, additives, and the additives–polymers mixture will help to extend the knowledge on the state of polymer degradation, establish the link between formulation, properties, and degradation [58], and target toxic aspects; thus, this technique should be optimized and used in microplastics monitoring.

## 4. Conclusions

The sampling of polychaete was carried out within one year, with a 5-month interval at four different sampling stations. The result indicated that the most abundant color of microplastics that was ingested by the polychaetes during the northeast monsoon season was a brown color, whereas those that are transparent in color were more preferable during the southwest monsoon. The main reason for this observation is that all samples were collected in the digestive system of polychaetes and the original color of microplastics could be decolorized due to the ageing process or photo-degradation, as well as the biological interaction in the polychaete guts. The microplastics were further analyzed by employing chemical characterization via pyrolysis-GC/MS techniques, and the data indicated that several polymers were detected in the sample, including PVC, PE, PP, PS, PA, PMMA, and PET. The application of Py-GC/MS enables information on the composition of the plastic material to be obtained, though without clear data on the formulation. Additionally, the possibility of obtaining false positive data cannot be eliminated since Py-GC/MS will eventually pyrolyze all possible materials, hence producing similar molecular structures to those monomers. Nevertheless, it is proven that this technique can be applied to the qualitative and semi-quantitative analysis of a large variety of additives in polymeric items. Furthermore, analytical pyrolysis provides an extensive benefit in analyzing a complex sample for most compounds. It can determine mixed media and analyze both the polymer and associated organic plastic additives content in both an analytical amount and small size of samples, such as samples of invertebrates and planktonic microorganisms.

## Figures and Tables

**Table 1:** Information of each sampling sites.

Sampling Sites	Latitude	Longitude
S1	5°43′21.48″ N	102°40′13.87″ E
S2	5°41′30.77″ N	102°41′54.98″ E
S3	5°40′54.46″ N	102°42′33.25″ E
S4	5°39’49.27″ N	102°43’57.69″ E

**Table 2:** Characteristic compounds for 12 polymers, their characteristics ions, and retention time.

Polymer	Characteristic Compound	Main Characteristic Ion (*m*/*z*)	Sub Characteristic Ion (*m*/*z*)	Retention Time (min)
PE	1,20-Heneicosadiene	82	294	15.427
PP	2,4-Dimethyl-1-heptene	126	70	6.052
PS	Styrene trimer	91	312	19.96
ABS	2-Phenethly-4-phenylpent-4-enentrile	170	91	17.17
SBR	4-phenylcyclohexene	104	158	11.016
PMMA	Methyl methacrylate	100	69	4.328
PC	4-Isopropenylphenol	134	119	10.696
PVC	Naphthalene	128	115	9
PU	4,4′-Methylenedianiline	198	106	17.25
PET	Benzophenone	182	105	13.608
N-6	Ɛ-Caprolactam	113	85	10.677
N-66	Cyclopentanone	84	55	5.637

**Table 3:** Identification of polymer associated with microplastics found in polychaete samples during the sampling period and stations.

Period and Station	1st				2nd				3rd				4th			
	S1	S2	S3	S4	S1	S2	S3	S4	S1	S2	S3	S4	S1	S2	S3	S4
PE	+	+	**-**	**-**	**-**	+	+	**-**	+	**-**	+	+	+	+	+	**-**
PP	+	+	+	**-**	+	**-**	+	+	+	+	+	+	**-**	+	+	+
PS	**-**	**-**	**-**	**-**	+	**-**	+	**-**	+	**-**	**-**	**-**	**-**	**-**	+	**-**
PVC	++	++	++	++	++	++	++	++	++	++	++	++	++	++	++	++
ABS	**-**	**-**	**-**	**-**	**-**	**-**	**-**	**-**	**-**	**-**	**-**	**-**	**-**	**-**	**-**	**-**
PET	**-**	**-**	**-**	**-**	+	**-**	+	+	**-**	**-**	**-**	**-**	**-**	**-**	**-**	**-**
PC	**-**	**-**	**-**	**-**	**-**	**-**	**-**	**-**	**-**	**-**	**-**	**-**	**-**	**-**	**-**	**-**
PMMA	+	+	**-**	**-**	**-**	+	+	**-**	+	**-**	**-**	**-**	**-**	**-**	+	**-**
N-6	**-**	**-**	**-**	**-**	+	**-**	+	+	**-**	+	**-**	+	**-**	+	+	**-**
N-66	**-**	**-**	**-**	**-**	**-**	**-**	**-**	**-**	**-**	**-**	**-**	**-**	**-**	**-**	**-**	**-**
PU	**-**	**-**	**-**	**-**	**-**	**-**	**-**	**-**	**-**	**-**	**-**	**-**	**-**	**-**	**-**	**-**
SBR	**-**	**-**	**-**	**-**	**-**	**-**	**-**	**-**	**-**	**-**	**-**	**-**	**-**	**-**	**-**	**-**

“+” refers to detected, “++” refers to much detected”, and, “-“ refers to undetected.

## Data Availability

Not applicable.

## Supplementary Materials

The following supporting information can be downloaded at: https://www.mdpi.com/article/10.3390/polym14153054/s1, Figure S1: Prepared calibration curves for the analysis of twelve common polymers using Py-GC/MS.
