# Supplementary figures and images for: Utilizing Pyrolysis–Gas Chromatography/Mass Spectrometry for Monitoring and Analytical Characterization of Microplastics in Polychaete Worms
Source: Polymers (Basel). 2022 Jul 28;14(15):3054. doi: 10.3390/polym14153054 (PMC9370765; doi:10.3390/polym14153054)

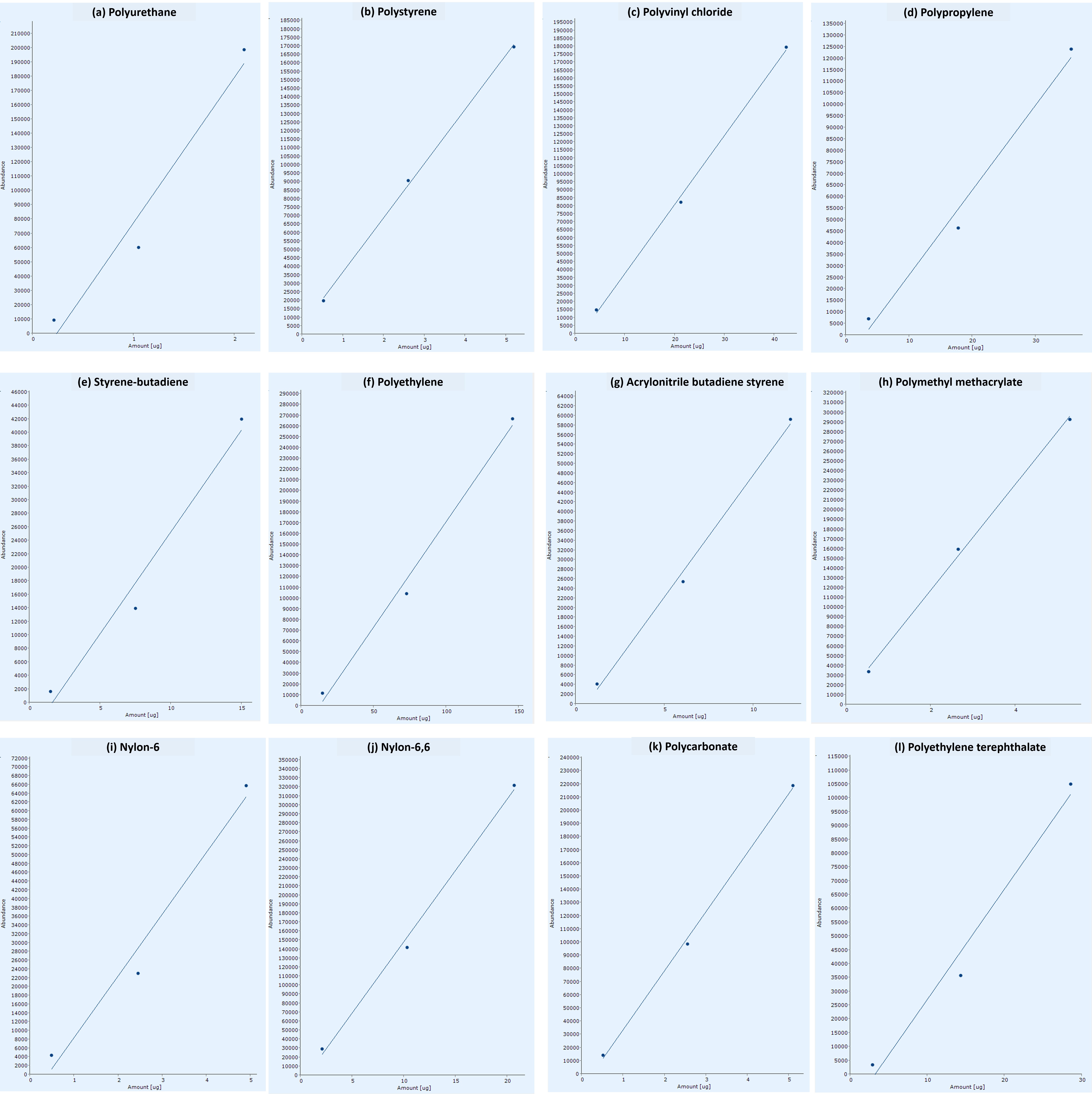

Supplement: Supplementary file 1 [file polymers-14-03054-s001.zip › polymers-1756714-supplementary.png]
